# T-ray Wavelength Decoupled Imaging and Profile Mapping of a Whole Wafer for Die Sorting and Analysis

**DOI:** 10.3390/s23073663

**Published:** 2023-03-31

**Authors:** Anis Rahman

**Affiliations:** Applied Research & Photonics, Inc., T-Ray Division, 470 Friendship Road, Suite 10, Harrisburg, PA 17111, USA; a.rahman@arphotonics.net; Tel.: +1-717-623-8201

**Keywords:** T-ray cameraless imaging, whole-wafer imaging, on-wafer die sorting, profile mapping, criterion for good die, high resolution with larger wavelength

## Abstract

This paper describes a terahertz (T-ray) cameraless imaging and profile mapping technique for accomplishing the imaging and/or mapping of a whole wafer with fabricated dies for devising a criterion to sort out good dies. A stratagem for decoupling the wavelength’s dependence on image formation is described, whereby the Abbe diffraction limit is overcome, and a high-resolution image is generated by a larger wavelength T-ray. The mechanics of cameraless image formation is discussed. A 200 mm diameter patterned wafer’s image details have been presented from which die-to-die inconsistencies were investigated. A profile of a row of dies was formed from the scanned intensity and compared with the profiles obtained from the graphical analysis of the image of the same dies. It is demonstrated that a criterion could be established either from the scanned profile or from the profile generated from the graphical analysis of the image. A known good die’s profile could be used as a reference to compare with the other dies’ profiles on the same wafer. Such a criterion could be used to sort the good from bad dies. The technique is extended to a whole wafer populated with die patterns via the profile mapping of the entire wafer. The profile mapping of the whole wafer could be used to compare and sort all wafers from the same batch. The Fab yield is improved by maximizing the count of good dies by applying the efficient sorting criterion.

## 1. Introduction

Billions of wafers are fabricated each year at foundries (Fabs) around the world. While the Fab process for a given IC is perfected as much as possible before mass production, it is known that, in some cases, the rejection rate of fully processed wafers is as high as 30%, especially for the 300 mm wafers with newer process nodes. Since the dies on patterned wafers must be singularized and packaged, it is of paramount importance to make sure that the dies are functional before going through the laborious and expensive task of packaging. An entire wafer is rejected when the percentage of defective dies exceeds a threshold. Obviously, the yield is directly dependent on the number of functional dies; therefore, defective dies must be identified and eliminated.

There exists a significant body of literature for modeling and predicting the wafers’ yield. The reported literature includes techniques such as wafer line productivity optimization [1] and a defect cluster index [2]. Other techniques and models have been adapted for yield analyses, including Murphy [3] and negative binomial [4] methods. The yield for semiconductor fabrication depends on many factors, including contaminants, inclusions, interface defects, delamination at layers, the accuracy of manufacturing instruments, the control of process parameters, and the design of semiconductors. As such, measurements (or metrology) at each step of the process flow are required. Finally, when the fabrication process is completed (i.e., the entire wafer is populated with dies), individual dies are singularized, electrodes are connected, and the chips are packaged for use on the final circuit board. Therefore, it is easily hypothesized that, after fabrication, all good dies will exhibit an identical profile along the same coordinates. That is, a line profile on a given good die along a given coordinate will match the profiles along the same coordinates of other good dies.

This paper describes the cameraless T-ray imaging of a whole wafer, whereby the health (functionality) of the individual dies could be assessed before the laborious tasks of singularization and packaging. Here, a new instrument from Applied Research & Photonics (Harrisburg, PA, USA), the terahertz nanoscanning spectrometer and 3D imager (TNS3DI), was utilized for the imaging and profile mapping of a whole wafer with patterned dies on it. Both the scan profile and the profile obtained from a graphical analysis of the T-ray image were carried out, thus allowing a comparison of the dies with respect to each other. The profile of all dies along a given line is expected to be identical if they possess identical morphology and/or topology. The profile of a known good die, therefore, is important and should be used as a reference. The profiles of other dies are compared to the reference and a criterion of go/no go for a specific die has been devised. In addition, a profile map of the whole wafer has been proposed, whereby all wafers in each batch may be sorted with respect to the profile map of a known good wafer with the dies of the same chips.

The main motivation for using the T-ray for the proposed die sorting stems from the extraordinary sensitivity available from this approach. While IR or visible light is used in many current techniques T-ray’s sensitivity is unparalleled. IR, for example, is sensitive to the bond vibrations that are used for molecular identification via Raman spectroscopy. The T-ray, on the other hand, is sensitive to many more molecular resonances, such as vibration, translation, rotation, torsion, etc. Thus, while IR is sensitive to the bonds, the T-ray is sensitive to all kinds of resonances present in a molecule. This sensitivity yields capabilities beyond spectroscopy, offering other applications where critical identification is needed, such as the one discussed in the present paper.

## 2. Materials and Methods

### 2.1. Mechanics of Cameraless Image Formation: Decoupling the Wavelength

Cameraless imaging details have been described elsewhere [5]. A brief description is given here. The main objective for avoiding the camera route is twofold: first, to overcome the Abbe diffraction limit and, second, to achieve Angstrom scale resolution with larger wavelengths, such as the T-ray. Since the camera route depends on a charged coupled device (CCD) or a similar focal plane array, the resolution of a camera is entirely dependent on the resolution of such a device. The same is true for any image-recording instrument. The Abbe diffraction limit [6] established an upper boundary of resolution achievable by any electromagnetic (EM) energy; the smallest object that can be resolved is half the wavelength of the light (EM energy) being deployed for imaging. Since the electron wavelength is in picometers, an electron microscope can resolve the atomic lattice. Therefore, any technique deploying the wavelength-based image formation must obey the Abbe diffraction limit; hence, visible light and/or UV light cannot image the atomic lattice. Scientists had thought that if one could overcome this physical barrier of the Abbe diffraction limit, then imaging the lattice with a larger wavelength could have been possible. However, this connotation was proven wrong, because as long as wavelength-dependent imaging is utilized, the diffraction limit is unavoidable. Therefore, the decoupling of wavelength dependence on image formation is the key to achieving higher image resolutions with larger wavelengths. At the same time, to be versatile, such a technique should not only be limited to resolving smaller objects (e.g., atomic lattice) but it should also be able to image larger objects at the macroscopic size (macroimaging). This paper discusses such a technique of wavelength-decoupled imaging that is applicable to both lattice-scale imaging and macro-scale imaging. Here, a whole wafer has been imaged to demonstrate the macroimaging method while epitaxial semiconductor layers have been analyzed by lattice scale imaging [7] and lattice dilation [8] via the same technique, as implemented in a single instrument.

**Figure 1:**
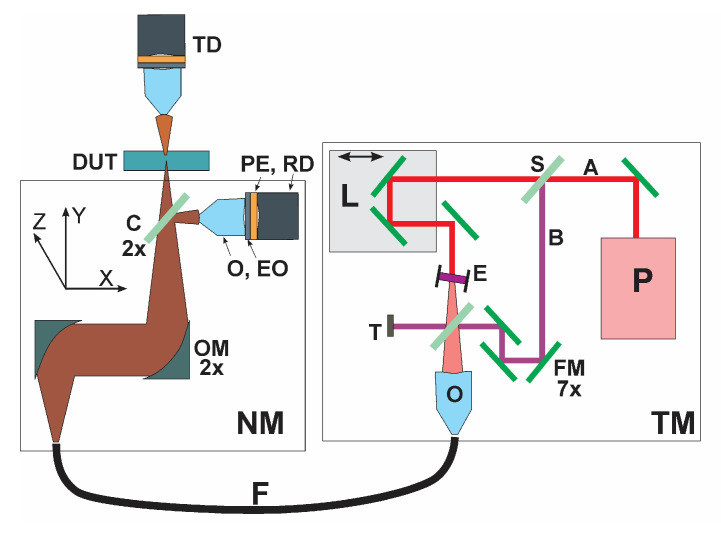
The optical circuit is mounted on an XYZ nanoscanner (not shown). Both reflection mode and transmission mode measurements are possible. Here, the sample remains stationary while the nanoscanner will scan the sample over a chosen area or volume. Adapted from [5]. Legends: P: Pump laser, L: Delay line, S: Beam splitter, A: Pump beam, B: Probe beam. E: T-ray emitter, FM: Fixed mirrors (7x), C: Beam combiner (2x), OM: Off-axis mirror (2x), O: Objective (3x), EO: Electro-optic sensor, PE: Polyethylene filter (2x), RD: Detector (reflection), TD: Detector (transmission), T: Beam stop, DUT: Device under test, TM: Terahertz module, NM: Nanoscanner module.

The principle of decoupling the wavelength dependence of the image’s formation is briefly described here. Decoupling is accomplished via an indirect image formation stratagem where a two-step procedure is deployed. Since all image formation takes place in reflections, we devised a technique for scanning an object via T-ray reflections. This is the first step. That is, an object is scanned over a volume by focusing the T-ray on it at a vertical incidence in a non-contact fashion. The wafer (or a sample) remains stationary while the T-ray beam scans it by means of a nanoscanner, as shown in Figure 1. The volume to be scanned is selected by the user, and the scanning resolution is also user selectable. The current nanoscanner allows a scan speed of 500 nm/s on the lowest setting, ~10 mm/s on the highest setting, and anything in between as selected by the user. The lowest speed is deployed for lattice resolution imaging while a higher speed is selected depending on the image resolution’s requirements. Higher scanning speeds correspond to a larger pixel size (or voxel size in 3D), i.e., lower resolution. A streaming data acquisition algorithm is implemented, where the data rate is independent of the scanning speed. This enables higher data density at lower speeds and proportionately lower data density at higher speeds. The wafer (sample) is scanned one surface at a time. The entire volume is divided into a number of surfaces depending on the step length chosen by the user. Each surface is scanned one at a time by adjusting the focal point on a desired surface or a subsurface, and the streamed reflected intensity is stored in a data file. The reflected intensity can be expressed in terms of a modified Beer–Lambert law, *R* = α*lϵ*, where *α* is the molar absorptivity, *l* is the path length, and *ϵ* is the dielectric constant [5] or any other physical constant of the material. Measured reflectance, *R*, is a material-dependent parameter and thus also depends on the position of the incident beam on the sample being imaged or profiled because the object (e.g., a semiconductor wafer) is made of different materials arranged in different patterns.

In summary, the decoupling of the wavelength from image formation physics was achieved by (1) scanning the object to be imaged by a nanoscanner, (2) storing the reflected intensity in a matrix termed the Beer–Lambert reflection matrix (BLR matrix), and (3) utilizing an algorithm to generate the volume image from the BLR matrix. The algorithm of the “inverse distance to power equation” was described in Ref. [5].

Once the image is generated, it can be rendered as a surface image or a volume image. The volume image can be sliced into any number of slices (surfaces) along all three orthogonal directions, thus allowing a layer-by-layer inspection of the sample along the *x*-, *y*-, and *z*-directions. The T-ray imaging technique as described here offers an important advent of defining one’s own pixel size (or voxel size in case of volume image) by a hardware and software combination, as opposed to being limited by the image sensor chip, such as the charged coupled device (CCD).

The pixel size (or the voxel size in 3D): As explained elsewhere [5], a streaming data acquisition algorithm has been worked out where both the reflected intensity and the stage positions are read continuously while the stage moves at a speed specified by the user. The measured steps (location of the stage) are used as the position parameter in generating the image, but the pixel size (or the voxel size) is not the same as the step size (see below). The *x*-axis step size (hardware step) is set by the stage speed. Slower speeds yield smaller step sizes, and faster speeds yield larger step sizes. As indicated above, a stage can move as slow as 500 nm/s and as fast as up to ~10 mm/s. The acquisition time, therefore, is directly dependent on the speed set by the user. The *y*- and *z*-axis step size is directly entered into the program and not dependent on the speed.

The reflected intensity data are stored in an *n* × 4 matrix, where *n* is the number of rows corresponding to a line scan over the specified length, and the 4 columns correspond to the 3 position vectors, X-, Y-, and Z-, and the intensity vector, V. The matrix is then subjected to the inverse distance to power equation algorithm for image generation [5]. This is a technique used for grid-based image creation from measured {X⇀, Y⇀, Z⇀, and V⇀} data sets (i.e., the BLR matrix). Practical {x, y, z} data sets typically comprise irregularly spaced values. As such, it requires further computation to generate a grid-based map (also known as lattice for image). The gridding process effectively interpolates data values for the lattice that creates the image. In doing so, the algorithm accomplishes two important objectives. First, it decouples the image formation from the wavelength dependence, that is, it overcomes the diffraction limit. Second, it generates the pixel (or voxel) to represent the actual object being imaged by decoupling the step-size dependence. Note that this process is different than that of topography or tomography where the step size is translated to the pixel size. The interpolation via the inverse gridding method is used to generate an image at 1 Å resolution or less, thus eliminating the wavelength-dependent diffraction limit for image formation.

Recently, there have been proposals for artificial intelligence (AI) and machine learning (ML)-enabled optical imaging methods. However, as argued elsewhere, visible light is not as sensitive as T-rays, and it is not able to penetrate opaque substrates such as semiconductors, which is also the case for infrared light. While X-rays can penetrate many substrates, it lacks sensitivity, and it imparts ionizing energy, which may perturb the delicate circuitry. Therefore, the T-ray approach proposed here is the only means for achieving the high-sensitivity probing of dies on the entire wafer in a non-invasive and non-contact fashion.

### 2.2. Experimental

**Figure 2:**
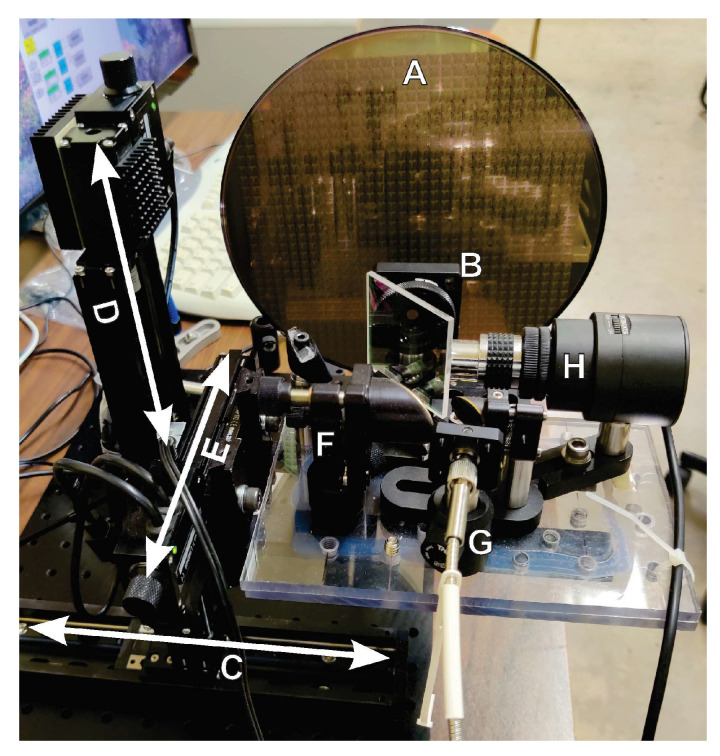
Nanoscanner setup for non-contact scanning T-ray beams and a stationary wafer mounting system. Any sample may be mounted and scanned over a user-defined volume. A: A 200 mm patterned wafer. B: Aperture for T-ray beam control. C, D, E: Nanoscanner axes: C: *x*-axis, 200 mm. D: *y*-axis, 200 mm. E: *z*-axis, 50 mm. F: T-ray control circuit. G: Fiber-coupled T-ray delivery. H: Objective with the detection system.

Figure 2 exhibits the nanoscanner setup. The 200 mm wafer (A) is mounted separately and not as a part of the T-ray delivery circuit, which is attached to the nanoscanner. Thus, the wafer remains undisturbed while the T-ray beam scans it by means of the nanoscanner. A 200 mm × 200 mm × 50 mm nanoscanner was used for imaging an entire fabricated wafer with all dies residing on the wafer. The Z-stage (50 mm travel) is used for scanning the depth of a sample for volume imaging.

## 3. Results

### 3.1. Comparison of the T-ray Image of Die Features with Optical Images

**Figure 3:**
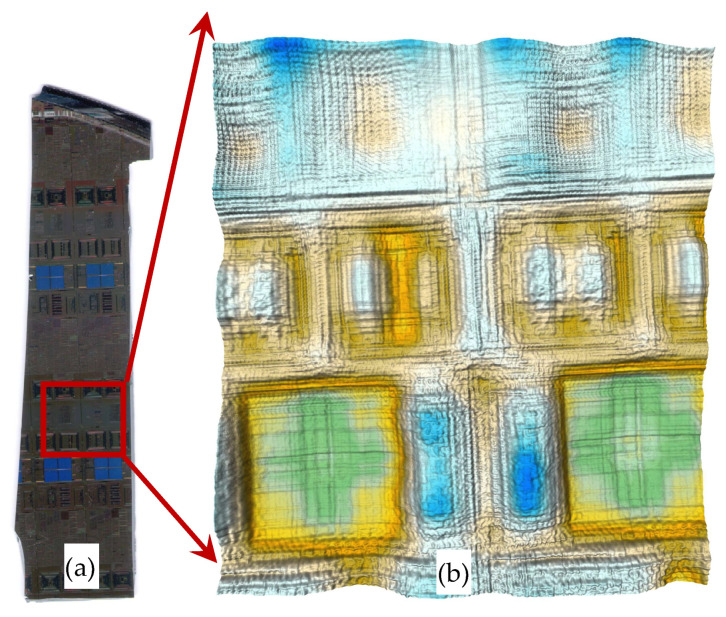
(**a**) Chip side of the wafer fragment’s optical image. (**b**) T-ray image (10 mm × 11 mm) corresponding to the rectangle shown in (**a**).

Here, a fragment of a patterned wafer has been imaged. The goal is to observe the pattern’s details along with its orientation. Therefore, the fragment was scanned by an optical scanner. The T-ray image is then compared side by side with an optical image. Figure 3a shows the fragment’s optical image obtained via a flat-bed scanner, and Figure 3b displays the T-ray image beside it for easy comparison. As observed, the T-ray image shows many details along with the identifiable orientation, which is important for aligning the dies.

### 3.2. Profile along a Row of Dies

**Figure 4:**
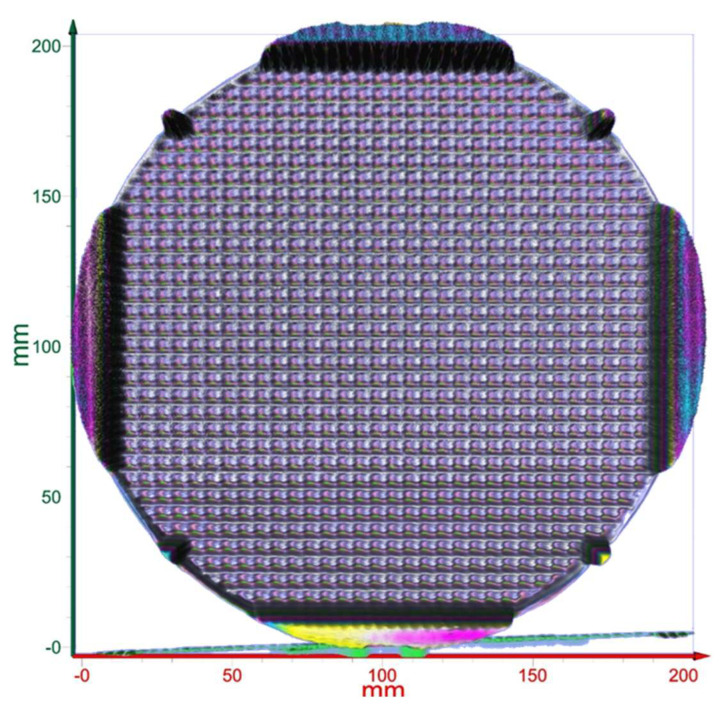
T-ray image of a 200 mm whole wafer with die patterns.

**Figure 5:**
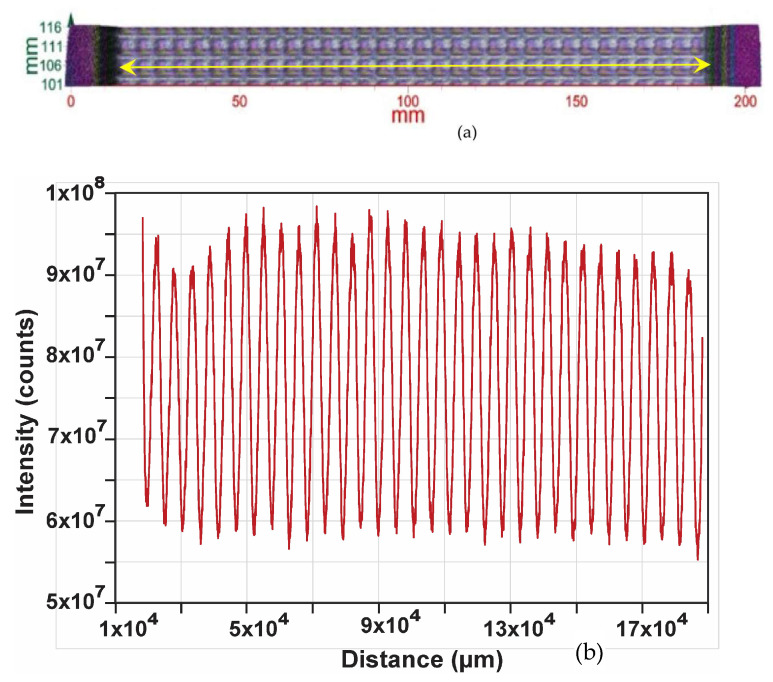
(**a**) shows 2 full rows of dies on the wafer shown in Figure 3, and (**b**) shows the scanned profile of 1 row at Y = 110 mm (i.e., around mid-point), as indicated by the yellow line in (**a**).

**Figure 6:**
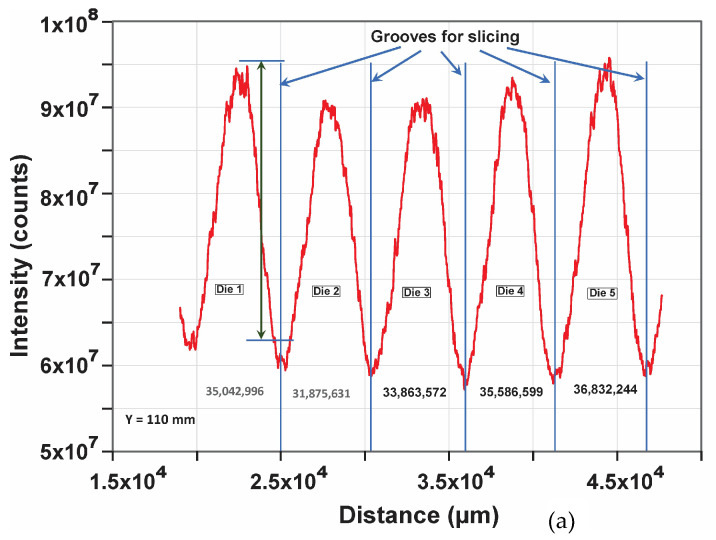

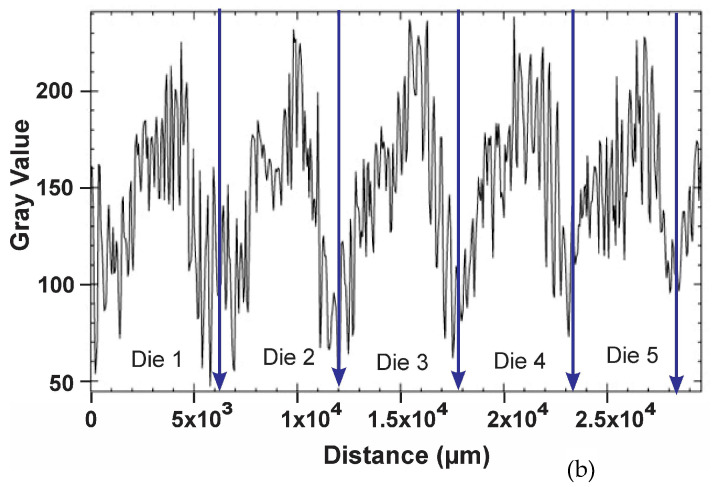
(**a**) Close up of the scanned profile of 5 consecutive dies extracted from Figure 5a. Profile detail of individual dies is visible. (**b**) Profile obtained via a graphical analysis of 5 consecutive dies from Figure 5a. The blue bars correspond to the groove positions for slicing.

We now consider a single row of dies anywhere on the wafer. Obviously, the number of dies is greater in the mid-region and increasingly lower towards either ends; therefore, we take a row in the mid-section of the wafer, as shown in Figure 4. Figure 5a exhibits an image of two full rows of dies on the whole wafer shown in Figure 4. Figure 5b exhibits the scanned (measured) profile of one row at Y = 110 mm (i.e., around the mid-point), as indicated by the yellow line in Figure 5a. As observed in Figure 5b, the measured profile of the row of dies in Figure 5a exhibits a regular pattern corresponding to the individual dies, with the exception that the reflected intensity of the dies shows some variations in magnitude. This variation is presumably due to some warp and/or thickness variation in the whole wafer, which is usually within the tolerance range of a given Fab process and not a likely reason for the die-to-die magnitude difference. The regular periodicity of the profile indicates that the dies are regularly spaced. However, the magnitude difference between the individual dies lying on the same plane, which is not due to the warpage, is indicative of some forms of die-to-die discrepancies. This is highlighted in Figure 6a for five consecutive dies. The fine features in the rise and fall of the profile are also meaningful. That is, in addition to the magnitude, these fine features in the profile are expected to be identical for identical dies. Thus, the profile as a whole of a known good die can be used as a metric for comparing and sorting other dies on the same wafer, as discussed below.

### 3.3. A Criterion for Sorting Good Dies

Referring to Figure 6a, the magnitude is defined by the difference in the intensity from the peak to the trough at a given (fixed) location on a die: magnitude = peak intensity—trough intensity (see the blue arrows in Figure 6a). The die-to-die magnitude variation is indicative of discrepancies between the dies. A criterion may be formulated to compare all dies with respect to the magnitude of a known good die.

Since the reflected intensity depends on the arrangement of materials on a die, specific locations are chosen on a die where the intensities from a line scan along these locations will produce the highest peak and the lowest trough, thus yielding the highest magnitude. Following this procedure, the intensity profiles of all dies are recoded by a computer program. After this first step, the disagreement between the magnitude of an individual die’s profile with respect to the reference die’s profile, Δ, could be computed:

(1) $$\Delta_{i}=\left|M_{ref}-M_{Di}\right|,$$

where $M_{ref}$ is the magnitude of the reference die, and $M_{Di}$ is the magnitude of the *i*th die. Ideally, all instances of $\Delta_{i}$ are expected to be identical. In practice, the lithography process may create some differences between the same features of different dies without significant deviation from functionality. It is, therefore, hypothesized that such differences will be small for a given die compared to the reference, and an acceptable limit may be established beyond which the functionality of a given die will deteriorate. That is, for a good die, a criterion can be set as,

(2) $$\Delta_{i}\leq \Delta_{max}=\left|M_{ref}-M_{Di}\right|$$

where $\Delta_{max}$ is the maximum acceptable value of the magnitude discrepancy beyond which a die must be eliminated from further packaging. For example, if one considers the Die 1 profile in Figure 6a as the reference, then $M_{ref}$ = 35,042,996, and the magnitude differences for Die 2 and Die 3 are $M_{2}$ = 31,875,631 and $M_{3}$ = 33,863,572, etc., respectively. If one assumes that $\Delta_{max}$ must be within 5% or less, then the measured $\Delta_{i}$ for each die on the present wafer must be within $M_{ref}\pm 5\%$. Thus, for the present case of Die 1—being the reference, albeit hypothetical—it, and the profiles of other dies compared to it, must be within 5% to qualify for further packaging. This is illustrated in Table 1 for the five dies shown in Figure 6a.

**Figure 7:**
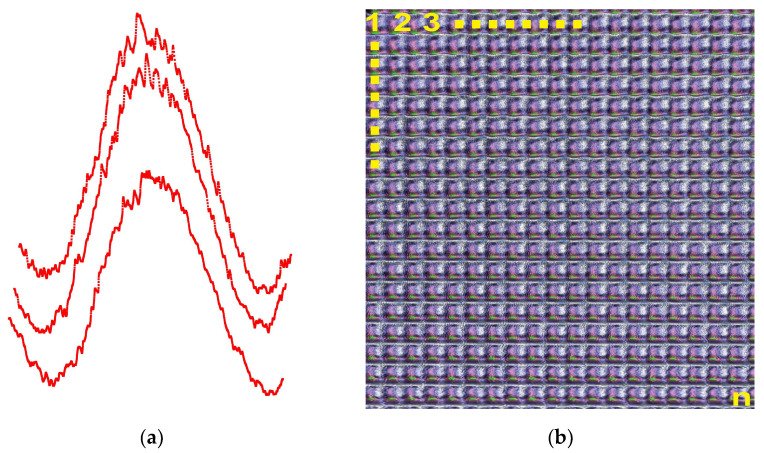
(**a**) Comparison of profiles of 3 dies for sorting criterion. (**b**) Image of all dies numbered for comparison and identification on a segment of a 200 mm patterned wafer.

The key factor in this magnitude-matching criterion is the measurement of the reflected intensity profile on the identical location of each die. Another option is pattern matching for each die profile. In addition to the matching of the magnitudes of die profiles, the entire profile must also be identical with that of the reference die. This is illustrated in Figure 7a. Here, the profiles of three consecutive dies from three rows are exhibited side by side (slightly spaced for clarity). Each profile is expected to fall on top of each other with respect to the reference die profile. Any deviation is indicative of discrepancies from the reference (good) die. Therefore, numbering the dies on the wafer (Figure 7b) and comparing the respective profiles with the reference profile allows an effective die sorting criterion that can be accomplished at a rapid speed.

**Figure 8:**
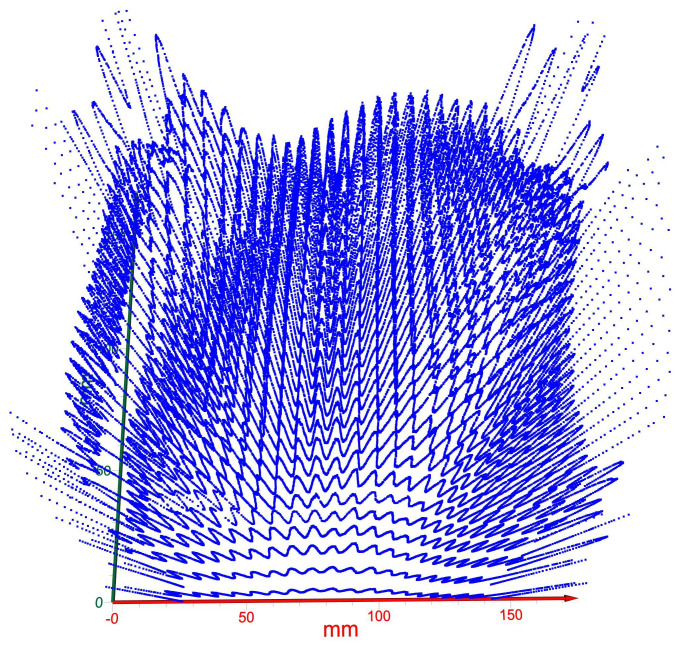
Scanned profile map created from profiles along a line on a row of dies for the entire wafer. This profile map is used for comparing the entire wafer with respect to a known good wafer.

This concept could then be extended to a whole wafer. Here, a known good whole wafer’s profile, similarly to the one shown in Figure 8, would be used as the reference that will be compared to the whole-wafer profiles of other patterned wafers from the same batch. This technique, thus, will speed up the sorting of good dies on a whole wafer and good patterned wafers from the same batch. The yield is defined by the ratio of the total number of functional dies on a wafer to the total number of dies designed per wafer. Hence, saving good dies that will otherwise be rejected will improve the yield significantly.

**Figure 9:**
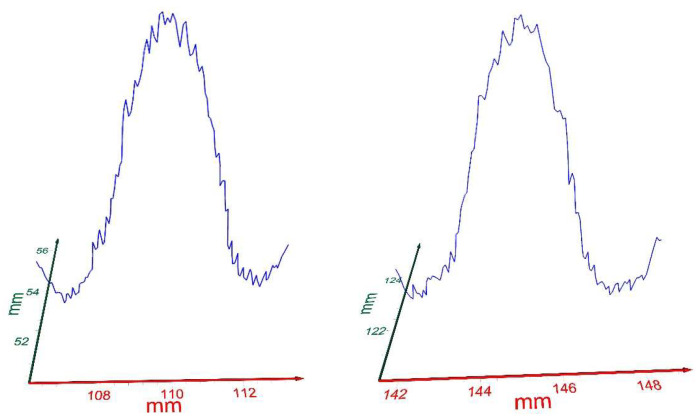
Profiles of a pair of dies from two different (XY) locations.

The T-ray profile mapping technique’s effectiveness is not affected by the warpage/bowing of a wafer. The warpage of a whole wafer is a macroeffect. The patterned individual dies lie on a local plane of their own, be it on an elevated location or a lower valley location of the wafer. That is, a single die has a material arrangement such that a line scan on a given die at known coordinates will produce an identical profile to a line scan on another die at the same coordinates, even though they are at two different physical locations on the wafer. This is true because the reflected intensity of each material is a unique property of its own, as illustrated in Figure 9. The minute variations in each deposited/patterned materials due to the lithography process are accurately detected by the T-ray, while other radiations are not able to accomplish this because of their sensitivity limitations. Figure 9 exhibits the profiles of a pair of dies from two different (XY) locations of different elevations due to the warpage of the wafer. In view of the profiles of these two dies (Figure 9), it is evident that the local profiles of both dies are similar regardless of the warpage of the wafer; thus, the proposed criterion (Equation (2)) should be applicable, as exemplified in Table 1.

## 4. Discussion

The technique introduced in this paper has a few components. First, there is the cameraless imaging of the whole wafer via T-ray nanoscanning, where the scanned intensity matrix (the BLR matrix) is converted to an image by an algorithm [9]. Second, the profiles of the dies are generated and recorded against each numbered die. Here, the profiles of each die are compared to a known good die. The dies are then accepted or rejected based on the criterion proposed. However, a known good die for a given process is only available under the actual Fab settings; therefore, an arbitrary die was taken as the reference as an example for the demonstration of the proposed criterion. The reference die will be easily chosen at the Fab by the operator by taking the profile of a functional die. Third, in some cases, the entire wafer may have some bow and/or warpage. It is known that semiconductor wafers are not perfectly flat. Wafers with larger dimensions, in particular, may have bowing/warping of a few micrometers across the entire wafer area, for which its acceptable range is determined before deploying a wafer to the Fab process. The profile map (Figure 8) shows such a case where the warping of the wafer is visible. However, the proposed profile mapping compares the profile of individual dies for sorting, where the measured magnitude difference (Equation (2)) of each die is not affected by the warp of the entire wafer. Since the magnitude is computed for each die individually, the wafer’s warpage has a minimal or negligible effect, and it is assumed that it should be contained within the proposed acceptable $\Delta_{max}$ of 5% or less.

For example, if a random die is selected by an operator during Fab production and is an outlier (bad die), then the criterion proposed may fail to identify the good dies. This is a very important point, and the success of the proposed criterion certainly depends on identifying a “good” die as the first step. Usually, Fab operators are experts in verifying if the reference die is indeed a good die. Given that, electrical testing will most likely be carried out to pin on a good die (or some other way as decided by a given Fab operator) before turning the wafer to the profile mapper. A machine learning and/or AI algorithm may also be implemented for an accurate choice at this step.

While it is a good idea to compare the proposed technique with other recent methods, our search did not produce any other T-ray-based or similar technique for an apple-to-apple comparison. For example, an online article says, “It takes numerous processes to complete a semiconductor chip, and testing to sort of defective chips is the final step. There are a number of tests carried out in the semiconductor manufacturing process. Electrical Die Sorting (EDS) is carried out when the wafer is completed, package testing is carried out after the chip is assembled and packaged, and final quality testing is carried out from the consumer’s perspective immediately before release” [10]. Another source says, “… die sorting and inspection system provides inspection before die assembly to help engineers quickly identify any issues during the dicing process of wafer-level packages and bare dies”. These sources show that this is a process after or during dicing and is not applied on the entire wafer [11]. Thus, it appears that there is no T-ray approach nor any other technique for sorting all the dies on an entire wafer.

## 5. Conclusions

This paper demonstrates a cameraless T-ray imaging technique for sorting good dies on a patterned wafer by comparing the scanned profiles of the dies with respect to a known good die’s profile. Comparing the profile along the chosen coordinates of a die with that of a known good die’s profile forms the basis for a rapid sorting criterion. The current art tends to reject an entire wafer if the number of defective dies exceeds a threshold without inspecting the rest of the dies from which some good dies may be identified. This practice is primarily carried out to save the time and effort of current laborious testing tasks. The T-ray imaging and profiling techniques described herein will allow the programmatic sorting of all dies on a patterned wafer and enumerate good dies for subsequent packaging. This procedure improves yields significantly.

The proposed procedure of sorting involves T-ray profiling a row of dies at a given location that is determined with respect to a known good die. The procedure is then extended to the entire wafer by profiling a known good wafer. The profile map of the known good wafer is then used as the reference to compare with the profile maps for the wafers from the same batch. With further development, the T-ray profile mapping system may be installed for the in-line inspection of wafers in a batch.

## Figures and Tables

**Table 1:** Criterion example summary.

#	*M*	D	%	Comment
Die 1	35,042,996	-	-	Ref
Die 2	31,875,631	3,167,365	9	Fail
Die 3	33,863,572	1,179,424	3	Pass
Die 4	35,586,599	−543,603	−2	Pass
Die 5	36,832,244	−1,789,248	−5	Pass

## Data Availability

The data supporting reported results may be obtained from the author. Please email a.rahman@arphotonics.net with requests.

## References

[1] Maynard D.N., Rosner R.J., Kerbaugh M.L., Hamilton R.A., Bentlage J.R., Boye C.A. Wafer Line Productivity Optimization in a Multi-Technology Multi-Part-Number Fabricator. Proceedings of the IEEE/SEMI 1998 IEEE/SEMI Advanced Semiconductor Manufacturing Conference and Workshop (Cat. No.98CH36168).

[2] Chao L.-C., Tong L.-I. (2009). Wafer defect pattern recognition by multi-class support vector machines by using a novel defect cluster index. Expert Syst. Appl..

[3] Murphy B.T. (1964). Cost-Size Optima of Monolithic Integrated Circuits. Proc. IEEE.

[4] Stapper C.H. (1973). Defect Density Distribution for LSI Yield Calculations. IEEE Trans. Electron Devices.

[5] Rahman A., Rahman A.K. (2019). Nanoscale Metrology of Line Patterns on Semiconductor by Continuous Wave Terahertz Multispectral Reconstructive 3-D Imaging Overcoming the Abbe Diffraction Limit. IEEE Trans. Semicond. Manuf..

[6] Abbe E. (1873). Beiträge zur Theorie des Mikroskops und der mikroskopischen Wahrnehmung. Arch. Mikrosk. Anat..

[7] Rahman A. Terahertz Multispectral Imaging of Epitaxially Grown Semiconductors’ Lattice Defects. Proceedings of the 2017 28th Annual SEMI Advanced Semiconductor Manufacturing Conference (ASMC) 2017.

[8] Rahman A., Tanzella F., Rahman A.K., Page C., Godes R. (2019). Lattice Dilation of Plasma Sprayed Nickel Film Quantified by High Resolution Terahertz Imaging. Nov. Res. Sci..

[9] Campregher N., Cheung P.Y.K., Constantinides G.A., Vasilko M. Analysis of yield loss due to random photolithographic defects in the interconnect structure of FPGAs. Proceedings of the 2005 ACM/SIGDA 13th International Symposium on Field-Programmable Gate Arrays—FPGA ’05.

[10] https://semiconductor.samsung.com/support/tools-resources/fabrication-process/eight-essential-semiconductor-fabrication-processes-part-8-eds-electrical-die-sorting-for-the-perfect-chips/.

[11] https://www.kla.com/products/packaging-manufacturing/die-sorting-and-inspection.

